# Performance improvement of machine learning techniques predicting the association of exacerbation of peak expiratory flow ratio with short term exposure level to indoor air quality using adult asthmatics clustered data

**DOI:** 10.1371/journal.pone.0244233

**Published:** 2021-01-07

**Authors:** Wan D. Bae, Sungroul Kim, Choon-Sik Park, Shayma Alkobaisi, Jongwon Lee, Wonseok Seo, Jong Sook Park, Sujung Park, Sangwoon Lee, Jong Wook Lee

**Affiliations:** 1 Department of Computer Science, Seattle University, Seattle, Washington, United States of America; 2 Department of ICT Environmental Health System, Graduate School, Soonchunhayang University, Asan, South Korea; 3 Department of Internal Medicine, Soonchunhyang Bucheon Hospital, Wonmi-gu, Bucheon-si, Gyeonggi-do, South Korea; 4 College of Information Technology, United Arab Emirates University, Abu Dhabi, UAE; 5 Department of Informatics, Technical University of Munich, Munich, Germany; Newcastle University, UNITED KINGDOM

## Abstract

Large-scale data sources, remote sensing technologies, and superior computing power have tremendously benefitted to environmental health study. Recently, various machine-learning algorithms were introduced to provide mechanistic insights about the heterogeneity of clustered data pertaining to the symptoms of each asthma patient and potential environmental risk factors. However, there is limited information on the performance of these machine learning tools. In this study, we compared the performance of ten machine-learning techniques. Using an advanced method of imbalanced sampling (IS), we improved the performance of nine conventional machine learning techniques predicting the association between exposure level to indoor air quality and change in patients’ peak expiratory flow rate (PEFR). We then proposed a deep learning method of transfer learning (TL) for further improvement in prediction accuracy. Our selected final prediction techniques (TL1_IS or TL2-IS) achieved a balanced accuracy median (interquartile range) of 66(56~76) % for TL1_IS and 68(63~78) % for TL2_IS. Precision levels for TL1_IS and TL2_IS were 68(62~72) % and 66(62~69) % while sensitivity levels were 58(50~67) % and 59(51~80) % from 25 patients which were approximately 1.08 (accuracy, precision) to 1.28 (sensitivity) times increased in terms of performance outcomes, compared to NN_IS. Our results indicate that the transfer machine learning technique with imbalanced sampling is a powerful tool to predict the change in PEFR due to exposure to indoor air including the concentration of particulate matter of 2.5 μm and carbon dioxide. This modeling technique is even applicable with small-sized or imbalanced dataset, which represents a personalized, real-world setting.

## 1. Introduction

Asthma is a major public health problem for health care systems worldwide because of its high prevalence and rising socioeconomic burden [1–3]. In addition, it is one of the primary care-sensitive conditions whose symptoms can be controlled by effective care management and prompt treatment [4].

Early intervention requires personalized patient-centered care in a service-oriented model. Recently, emerging technologies and tools, such as early alerts and continuous monitoring, have shown immense potential [5–10]. Avoidable exacerbations account for 63% of the total annual asthma costs [11,12]; thus, patient-centered, preventive approaches that predicts patients’ exposure to risk factors and evaluates corresponding likelihood of symptom change could help the health care industry or individuals to achieve significant economical savings.

One study that evaluated the time activity pattern in asthma patients showed that they spent almost 83% of their time indoors, mostly at home [13]. Because such a large amount of time is spent indoors, a proper indoor air quality control with advance prediction techniques can contribute to improving the symptoms of asthma patients [14–16]. Modeling the relationship between indoor air quality and the risk of asthma exacerbation is critical for forecasting individual patient’s risks. Machine learning algorithms are one promising approach to accomplish this task.

Although a large number of studies have reported the effect of exposure to ambient air on the risk of asthma exacerbation, data of individual patient-level analysis with indoor air-quality are inconclusive. A paradigm shift has occurred in the field of environmental epidemiological study with the convergence of large digital data sources and improved remote sensing technologies for indoor air quality monitoring and air purification, especially with clustered personalized individual patient data. Recently, various machine-learning algorithms have been reported to provide mechanistic insights about the heterogeneity of clustered data of patient symptoms to predict the occurrence of each patient’s symptom or for personalized treatment and prevention strategies.

However, little is known about the relative strengths and weaknesses of the different machine learning approaches or about the effectiveness of common techniques for the improvement of model accuracy. We conducted this comparative study to evaluate the performance of nine conventional machine learning techniques with the aim of improving the forecasting accuracy. The first improvement method applied was imbalanced sampling (IS). IS promotes the minority class, while avoiding overfitting to the training data [17]. Hence this method was used for improving classification models in the presence of imbalance distribution in training data. A main challenge of machine learning is the limited size of training data. In particular, deep learning requires a large amount of data to train models for generalization and hence high-quality prediction. This problem is acute in individualized asthma risk prediction where datasets for individual patients are frequently very small.

The second improvement method we proposed was a machine learning technique of transfer learning (TL) which can help overcome this problem by focusing on fine tuning a pre-trained model with a small amount of specialized training data. This technique has been shown promise in cancer screening by improving the accuracy of models classifying medical images [18]. Yet no such studies have been performed in the area of individualized asthma prediction with limited training data for each patient. Our proposed TL method is a neural network based learning technique where a model is trained through two phases, the first phase is a training for source model using population data and it is further tuned for target model using an individual patient’s data in the second phase.

To achieve our study objectives, we combined our database with a machine learning framework that uses an inference engine to model the association between exposure levels of daily indoor air and the daily peak expository flow rate (PEFR) of individual, adult patients with asthma.

## 2. Materials and methods

### 2.1 Study population and quantification of health symptoms

Twenty-five adult nonsmoking participants, aged 32–78 years living in Incheon, Bucheon or Seoul, were recruited from among adult asthma patients who had enrolled in our environmental health smart study with connectivity and remote sensing technologies (ESCORT) [19]. We used each participant’s daily PEFR records (n = 3,750) collected by doctors and medical practitioners at the Soonchunhyang University Bucheon Hospital in South Korea. The original study protocols related to our study were approved by the research ethics committee of the Soonchunhyang University (IRB No. 202001-BR-001-01); written informed consent was obtained from all participants. The patients’ PEFR values were collected twice per day from November 1, 2017, to May 31, 2018. The participants were asked to maintain an indoor air quality monitoring unit in their home and record their activities every 30 min in a diary.

### 2.2 Indoor air quality measurement

Our target indoor air quality variables were fine Particulate Matter of 2.5 μm (PM2.5), carbon dioxide (CO_2_), temperature, and humidity. The PM2.5 concentrations were measured in participants’ main living spaces by laser-light scattering sensors with 2 min interval. The performance of the selected PM2.5 sensors were compared to the US EPA Federal Equivalent Method and their outcomes have been described previously [20]. In South Korea, PM2.5 concentrations and CO_2_ are among the most important indoor risk factors. Monitoring of these two variables have been recommended due to their potential health effects, including carcinogenetic effect or ability to induce dizziness, respectively, and their high-temporal variability is affected by patients’ activity pattern (indoor cooking, ventilation, etc.). Each patient’s real-time indoor air quality level (every minute) was converted to a daily mean level to match with each patient’s daily PEFR measurements; the data was stored on our database server for subsequent exposure calculations.

### 2.3 Measurement of peak expository flow rate

One of the common indicators of asthma-related symptoms is PEFR. To interpret the severity or exacerbation of PEFR levels, population-level data obtain using a peak flow meter is often used [21] with the green, yellow, and red zones, as stated by the American Lung Association [22]. However, because of the large variability of the baseline PEFR among patients, a standardized, patient-specific cluster data should be used to avoid false positives or false negatives [22].

In this study, we applied our forecasts to a simplified version of individual-specific exacerbation zoning method proposed by Alkobaisi in 2019 [23] that classifies a patient’s exacerbation level based on their own historical distribution of PEFR values.

We used two segmentations that included “safe zone” PEFR values in the upper 80% of the patient’s personal historical PEFR distribution, and “red zone” PEFR values in the lower 20%. Using machine learning-based inference engines, we attempted to predict the instances at which the patient had a risk of entering the red zone, which would likely pose a medical emergency. As described by Alkobaisi [23], it is important for doctors and patients to analyze each patient’s PEFR distribution, as it relates to their personal health condition, and actively aim to modify the 80/20 cutoff on an individual basis.

The conventional machine learning techniques used in this study attempted to identify when each patient was at risk, i.e., “red zone,” by forecasting when their PEFR value would be below the established cutoff. Although the cutoff may have been established based on a lower threshold of each patient’s historical PEFR values, the probability that this value would reduce below the critical PEFR value, i.e., 20% of their daily mean PEFR, on any specific day depends on many factors, and can differ by 20% on any given day. Accordingly, the model’s estimated probability of a patient’s PEFR value reducing below the cutoff value can be used as a measure of a patient’s daily health risk for that day.

### 2.4 Machine learning techniques to asthma risk prediction

The models we analyzed in this study applied a classification-based framework to forecast patients’ PEFR severity levels. Each model attempted to classify the patients’ daily PEFR zone based on their exposure to indoor air quality and its PEFR value on the previous day. The nine classification techniques we included in our comparative study were as follows: (1) decision tree gini (DT-G), (2) decision tree entropy (DT-E), (3) random forest (RF), (4) support vector machine (SVM), (5) K-nearest neighbors (KNN), (6) gradient boosting (GB), (7) logistic regression (LR), (8) naïve Bayesian (NB), and (9) neural network (NN).

### 2.5 Improvement in predictive techniques with imbalanced sampling

One of the most common challenges in machine learning-based classification occurs when the case of interest (e.g., low PEFR values) is relatively rare [17]. This imbalance can make the classifier prone to a preferential focus on the majority class. Conventionally, imbalanced classes are managed with up- and over-sampling [24]. Determining how much sampling to apply is one of the challenges with sampling techniques. An over-sampling ratio must be chosen so as to promote the minority class, while avoiding overfitting to the training data. Similarly, an under-sampling ratio must be chosen so as to retain as much information about the majority class as possible, while promoting a balanced class distribution. Up-sampling can be used for avoiding overfitting issues in over-sampling and for promoting a balanced class distribution [17].

In this study, we used both over-sampling and up-sampling methods together, in the so-called imbalanced sampling (IS), to improve the performance of the nine classification techniques. As shown in Algorithm 1, we first split each patient’s dataset into training and testing data. Next, we up-sampled the training data by duplicating randomly selected instances of the minority class with replacements until the size of the minority and majority classes matched. We then over-sampled by doubling the total size of the resulting dataset.

**Algorithm 1** imbalanced Classifier (*D*_*train*_, *A*)

1: **Input**: *D*_*train*_ is a set of class-labeled training data tuples, *A* is a classification scheme

2: **Output**: a classifier

3: Method:

4: *D*_0_ *←* tuples labeled with *class*_0_, *D*_1_ *←* data tuples labeled with *class*_1_

{*class*_0_: tuples with PEFR value ≤ 20^th^ quantile, *class*_1_: tuples with PEFR value > 20^th^ quantile}

5: n_0_ ← |D_0_|, n_1_ ← |D_1_|

6: $D_{0}^{\prime }$ ← {} {$D_{0}^{\prime }$ will contain up-sampled and over-sampled tuples of *class*_*0*_}

7: $D_{1}^{\prime }$ ← {} {$D_{1}^{\prime }$ will contain over-sampled of *class*_*1*_}

8: **repeat**

9: $D_{0}^{\prime }$ ← add a data tuple *t* selected from *D*_0_ using random sampling with replacement

10: *m ←* |$D_{0}^{\prime }$|

11: **until** *m ≈ n*_1_ is attained

12: $D_{0}^{\prime }$ ← update $D_{0}^{\prime }$ with duplicates of each data tuple from $D_{0}^{\prime }$

13: $D_{1}^{\prime }$ ←duplicates of each data tuple from *D*_1_

14: $D_{train}^{\prime }\leftarrow D_{0}^{\prime }\cup D_{1}^{\prime }$ {$D_{train}^{\prime }$ is an imbalanced training dataset}

15: classifier *C* ← trainModel ($D_{train}^{\prime }$, *A*)

### 2.6 Neural network based Transfer Learning (TL)

With performance improvement using the IS method in conventional machine learning techniques, this study further investigated neural networks as a deep learning framework. A neural network (NN) is a model whose predictive formula is defined by a layered mathematical structure. Their power comes partially from the fact that this structure gives practitioners a straightforward way to create models that detect arbitrarily complex nonlinear relationships between dependent and independent variables. However, their “black box" nature, susceptibility to overfitting, and the empirical nature of model development in practice, limit their use in health prediction modeling [25,26]. The overfitting problem is particularly acute in health-related fields, in which the sizes of datasets are generally smaller than the average for NNs. Datasets in individualized health risk prediction are relatively small in comparison to the datasets in image processing. For example, a patient’s daily PEFR dataset collected for 6 months includes less than 200 data points.

The paradigm of transfer learning (TL) attempts to solve the problem of insufficient data [27]. Mathematically, the transfer occurs by creating a new model through small adjustments to a model that performs well on one task with the aim of fine tuning it to perform well on the present task of interest [28]. TL works best in situations similar to the present one in which there is insufficient data to train a model on the task of interest but there is sufficient data available to train a good model on a related task.

**Fig 1:**
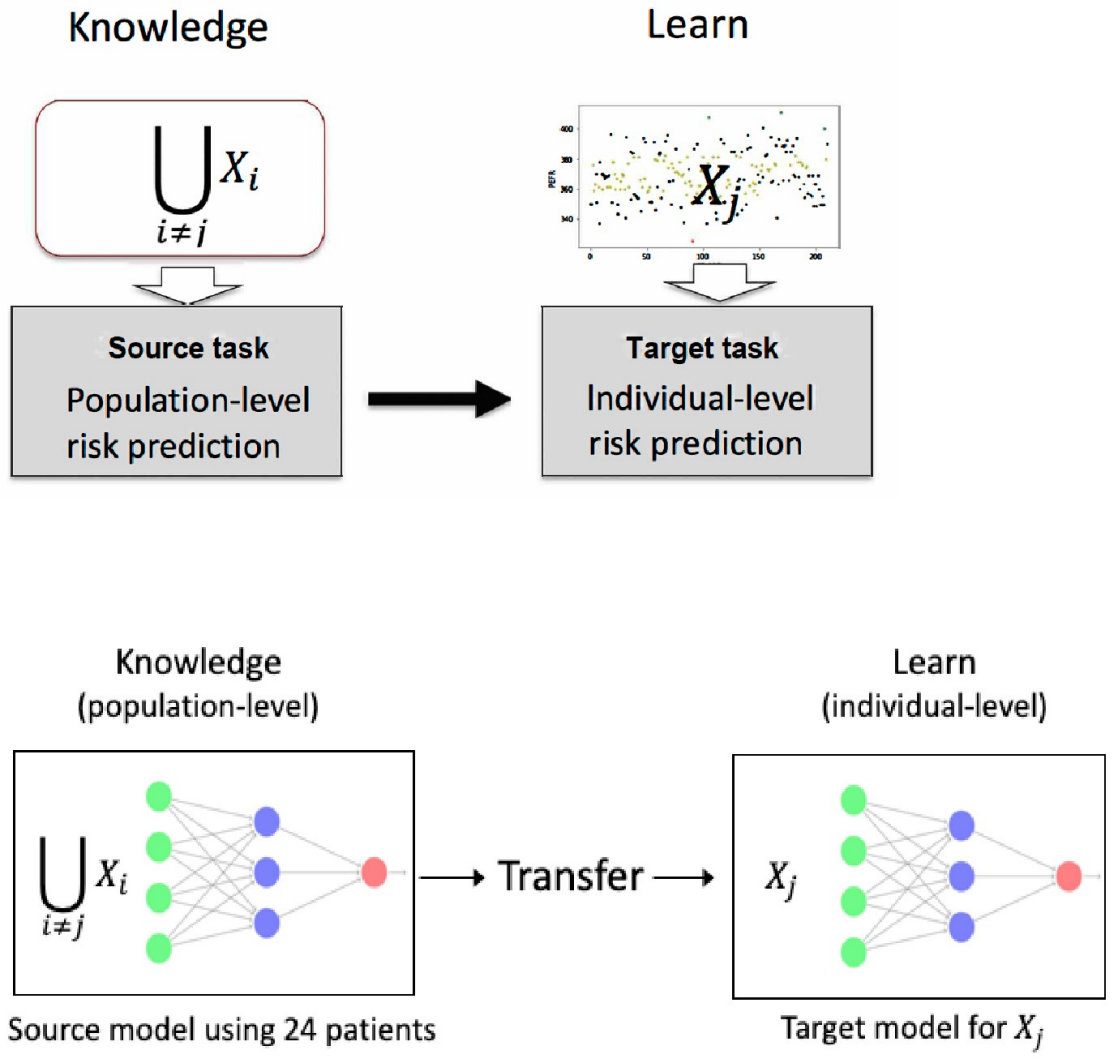
Outline of the methodology of transfer learning for risk prediction.

In this study of individualized asthma risk prediction, there was sufficient data to train a model to make good population-level predictions, but there was insufficient data to train an individualized model for each patient. The source task and the target task are the same; classify a patient’s next-day PEFR value as above or below their critical PEFR value. The source model was trained with 24 patients’ data (full population excluding the target patient’s data). The target model was then trained using the target patient’s data, as illustrated in Fig 1. In both source model and target model, we applied the imbalanced sampling method that was proposed for improving the overall quality of the predictive models.

**Fig 2:**
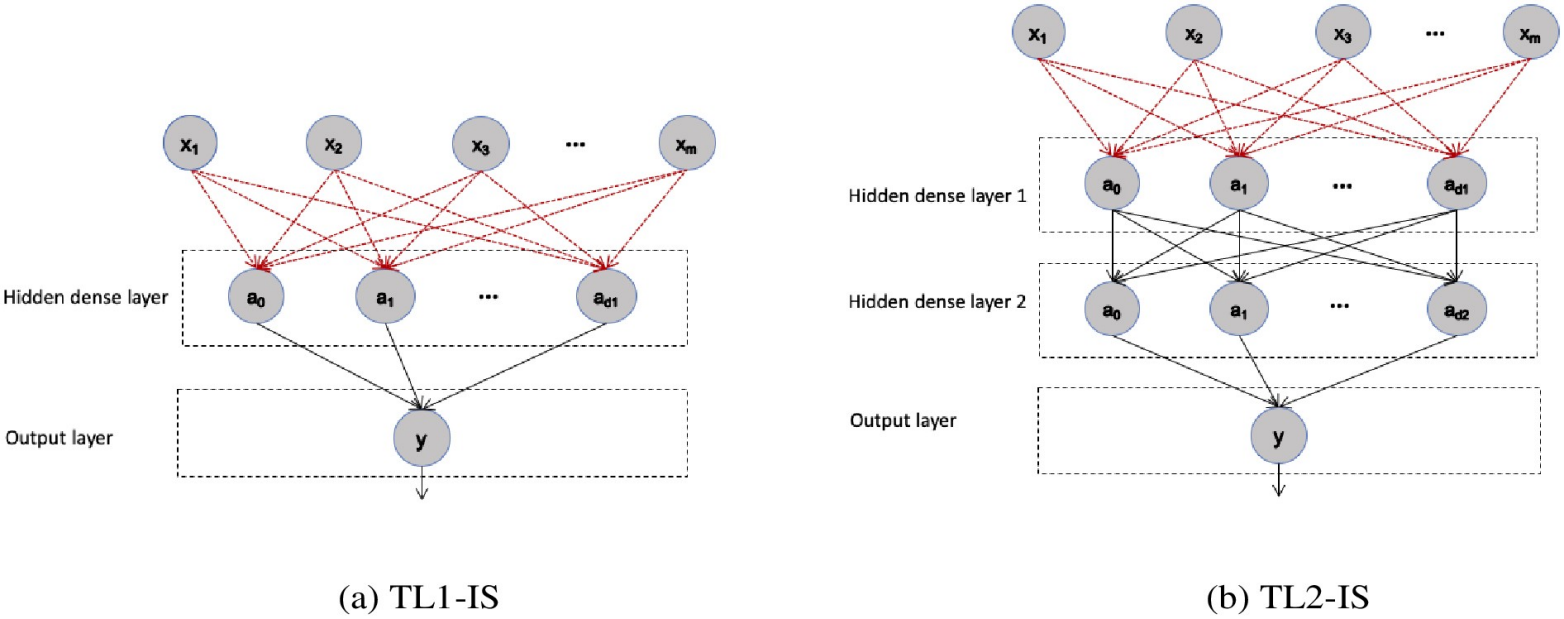
Transfer learning network topology. * Dotted lines represent frozen weights.

We present two architectures of transfer learning, TL1_IS for 1-hidden layer model and TL2_IS for 2-hidden layer model, and both models are trained with imbalanced sampling. To show the performance improvement through transfer learning, the two TL models were compared to a neural network with imbalanced sampling (NN_IS) that was trained only using the target patient data. In the TL modeling, we focus on the effect of freezing the weights of some initial layers of the source model and retraining only the later layers on the target data. All unfrozen layers for target models were initialized with the values from the source model. The model architectures evaluated together with what layers were frozen for the target model and other model information are given in Table 1. Fig 2 illustrates the two TL models’ network topologies studied in this paper.

**Table 1:** Neural network and transfer learning architectures.

model		Architecture	Unfrozen ratio	Total unfrozen weights
Neural Network	NN-IS	1 hidden layer	**100%**	321
		32 units		
Transfer Learning	TL1-IS	1 hidden layer	**10.3%**	33
		32 units (frozen)		
	TL2-IS	2 hidden layers	**79.1%**	1089
		32 units (frozen)– 32 units (unfrozen)		

* NN uses a target patient’s data only.

** TL uses 24 patients’ data except a target patient for source model and uses the target patient data for target model.

All of the networks studied are simple feed-forward neural networks with one or two hidden layers and an output layer returning a single value between 0 and 1 interpreted as the model confidence as to whether or not the patient’s next-day PEFR value will in the “red zone”. All model layers are fully connected with Rectified Linear Unit (ReLU) activation except for the output layer, which has sigmoid activation. All models were trained using Adaptive learning rate optimization (Adam) with binary cross entropy loss. The input layer of each model consists of 5 parameters, which are yesterday’s PEFR value and 4 indoor quality variables.

We trained the transfer learning models defined in Table 1 with 1000 epochs for the source model and 100 epochs for the target model. These values were taken from the optimal training durations found in the authors’ previous work with similar networks. The models were developed in Python 3.7 and Keras framework. The hyperparameters in the results were selected through extended training and validation processes to avoid over-fitting while increasing the accuracy. Each target model was evaluated through 3-fold cross validation giving 75 trained target models for each architecture. The process of the TL modeling is described in Algorithm 2.

**Algorithm 2** Transfer Learning(*X*, *X*_*j*_, *HP*_*learn*_, *HP*_*transfer*_)

1: **Input**: *X* is a set of class-labeled data tuples from *n*-1 patients, {X_1_,.., X_i_.., X_n-1_}, where *i ≠ j*, *X*_*j*_ is a set of class-labeled training data tuples of the target patient *j*

*HP*_*learn*_ is a set of hyperparameters for learning

*HP*_*transfer*_ is a set of hyperparameters for transfer

2: **Output**: performance metrics of a classifier

3: **Knowledge building process**:

4: Divide *X* into *k* datasets {for *k*-fold cross-validation}

5: **for** *i* = 1 to *k* − 1 **do**

6:  *X*_*train*_ *←* (*k −* 1) datasets

7:  *X*_*valid*_ *←* one remaining dataset

8:  a classifier *NN*_*learn*_ *←* imbalancedClassifierforNN(*D*_*train*_, *HP*_*learn*_)

9:  validateModel(*NN*_*learn*_, *D*_*valid*_)

10: **end for**

11: **Transfer learning process**:

12: **for** i = 1 to 3 **do** {for *3*-fold cross-validation}

13: *D*_*train*_ *←* 2/3 of *X*_*j*_

13: *D*_*test*_ *←* 1/3 of *X*_*j*_

14: a classifier *NN*_*transfer*_ *←* imbalancedClassifierforNN(*D*_*train*_, *HP*_*transfer*_)

16: performance metrics *←* validateModel(*NN*_*transfer*_, *D*_*test*_)

17: Report the average of performance metrics of 3-fold cross-validation

### 3. Results

**Fig 3:**
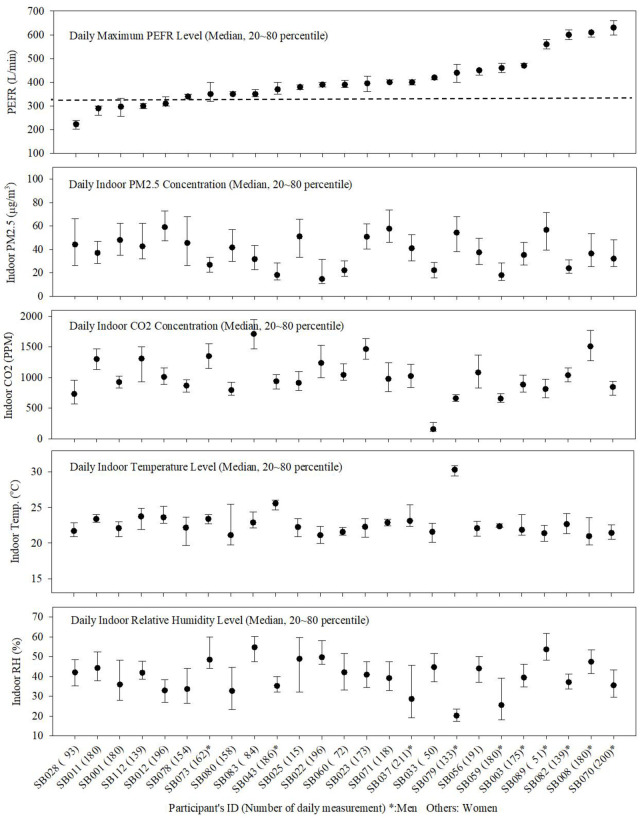
Distributions of participants’ daily peak expiratory flow rate (PEFR) levels, indoor PM2.5concentrations (μg/m3), CO2 (ppm), temperature (°C) and relative humidity (%) (The black dotted line in Fig 3 (top) represents the 20 percentile value (320 L/min) of overall 25 participants’ PEFR data).

We identified 25 patients who living within the study area and presented to the study hospital within the study period. Our final data comprised 3750 PEFR records (Table 2). Among our study patients, women were majority (60%); their median (interquartile range, IQR) age at the baseline was 58.0 years (50.0~66.0 years) and they were approximately 10 years younger than the men (68.5 years (59.0~74.0 years) but there was no statistical difference (p = 0.1067) (Table 2). Median (IQR) BMI was 23.8 (21.2~26.9) kg/m^2^ for female patients and 23.6 (22.7~24.0) kg/m^2^ for male patients (p = 0.9557). The median (IQR) of the PEFR of women was smaller than men (352 (310~400 L/min) and 470 (400~600 L/min), respectively) (p <0.001) (Table 2 and Fig 3). Indoor PM2.5 and CO2 concentration were higher (p<0.001) in women’s home than men’s one; (41.1 (28.5~54.7) μg/m3 vs. 31.8 (21.9~47.2) μg/m3; 1018.5 (847.8~1268.7) ppm vs. 926.2 (729.6~1178.4) ppm) (Table 2).

**Table 2:** Distribution of age, Body Mass Index (BMI), peak expiratory flow rate (PEFR) and indoor air quality (Temperature, Relative Humidity, PM2.5 and CO2) of studied participants.

	Overall (n = 25, 3750 episodes)			Women (n = 15, 2226 episodes)			Men (n = 10, 1524 episodes)			p-value* (W vs M)
	P50	P25	P75	P50	P25	P75	P50	P25	P75	
**Data size per patient**										
	**162**	118	180	**158**	104	185.5	**168.5**	134.5	180	0.8675
**Age (years)**										
	**60**	57	70	**58**	53	66	**68.5**	59.3	73.7	0.1067
**BMI (Kg/m2)**										
	**23.8**	21.5	25.6	**23.8**	21.2	26.9	**23.6**	22.7	24.0	0.9557
**PEFR (L/min)**										
	**390**	340	455	**352**	310	400	**470**	400	600	<0.001
**Temperature (**^**o**^**C)**										
	**22.6**	21.5	23.8	**22.6**	21.4	23.6	**22.7**	21.6	24.6	<0.001
**Relative Humidity (%)**										
	**39.7**	32.5	47.6	**40.6**	33.1	47.9	**38.3**	31.9	46.9	<0.001
**PM2.5 (μg/m3)**										
	**37.5**	25.2	52.4	**41.1**	28.5	54.7	**31.8**	21.9	47.2	<0.001
**CO2 (ppm)**										
	**984.9**	803.1	1248.4	**1018.5**	847.8	1268.7	**926.2**	729.6	1178.4	<0.001

Data size (# of days) per each patient: Minimum: 51, maximum: 200.

*p-value from Wilcoxon Mann-Whiney test.

### 3.1. Performance metrics of the predictive techniques

The performance of the classification techniques was evaluated for four quantities: true positive (TP), the number of positive data points correctly classified as positive; false negative (FN), the number of positive data points incorrectly classified as negative; false positive (FP), the number of negative data points incorrectly classified as positive; and true negative (TN), the number of negative data points correctly classified data as negative. Standard evaluation metrics that are defined in terms of these quantities are [24]: balanced accuracy, sensitivity, precision, and average F_1_-score. Given our focus on high-risk prediction, “positive” examples were those in which a patient’s PEFR value was below their critical cutoff (“red zone”). In the literature, this is often referred to as class_0_ and we adopted that convention herein. In the context of risk prediction, it was important to emphasize the model’s performance on the target class; therefore, balanced accuracy, sensitivity, and F_1_-score were good measures of a model’s ability to correctly predict a high risk of asthma.

**Fig 4:**
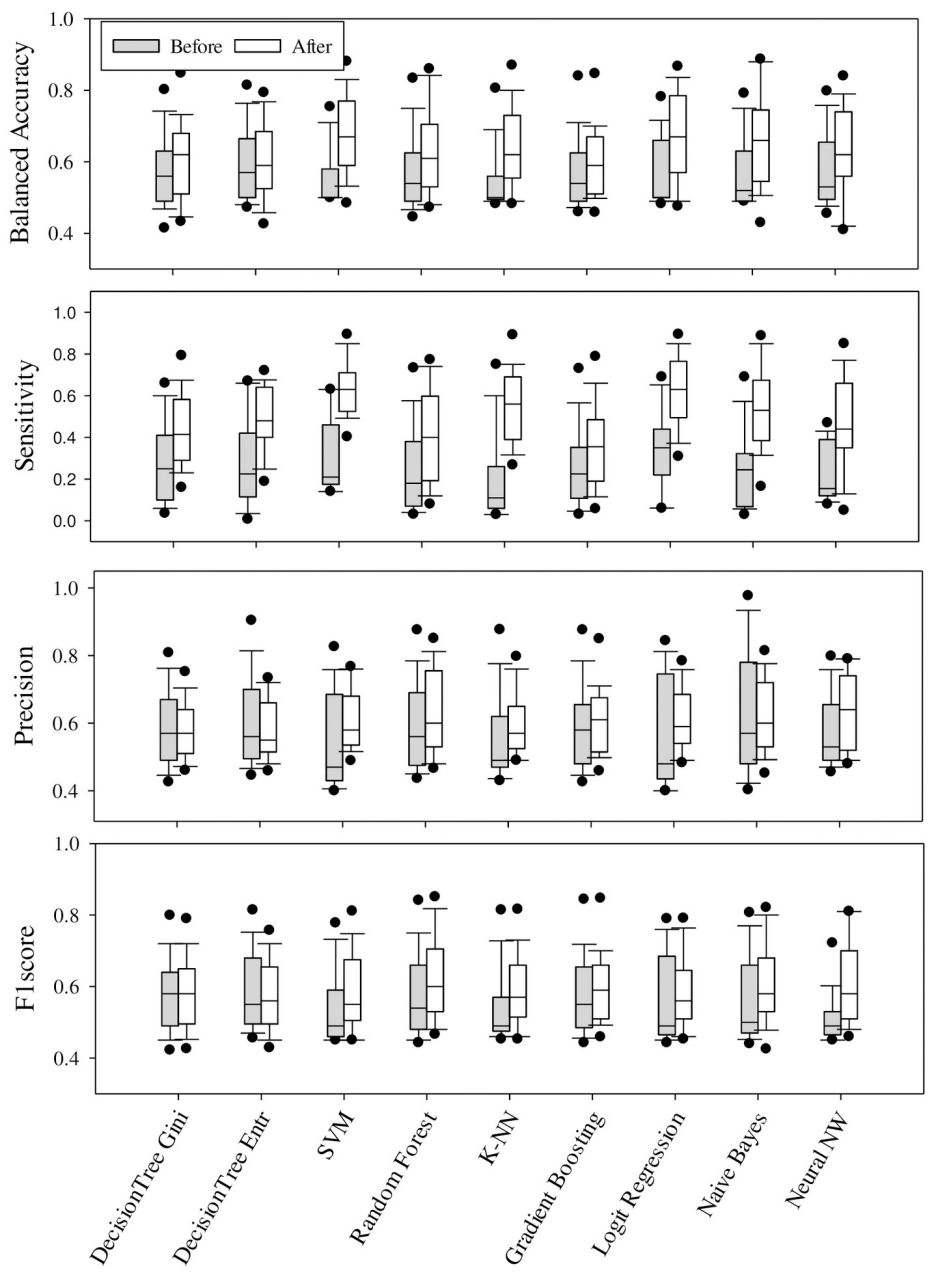
Performance results of nine predictive models (y-axis is generalized likelihood between 0.0 and 1.0; Gray box—Without imbalance sampling, White box—With imbalance sampling technique).

**Fig 5:**
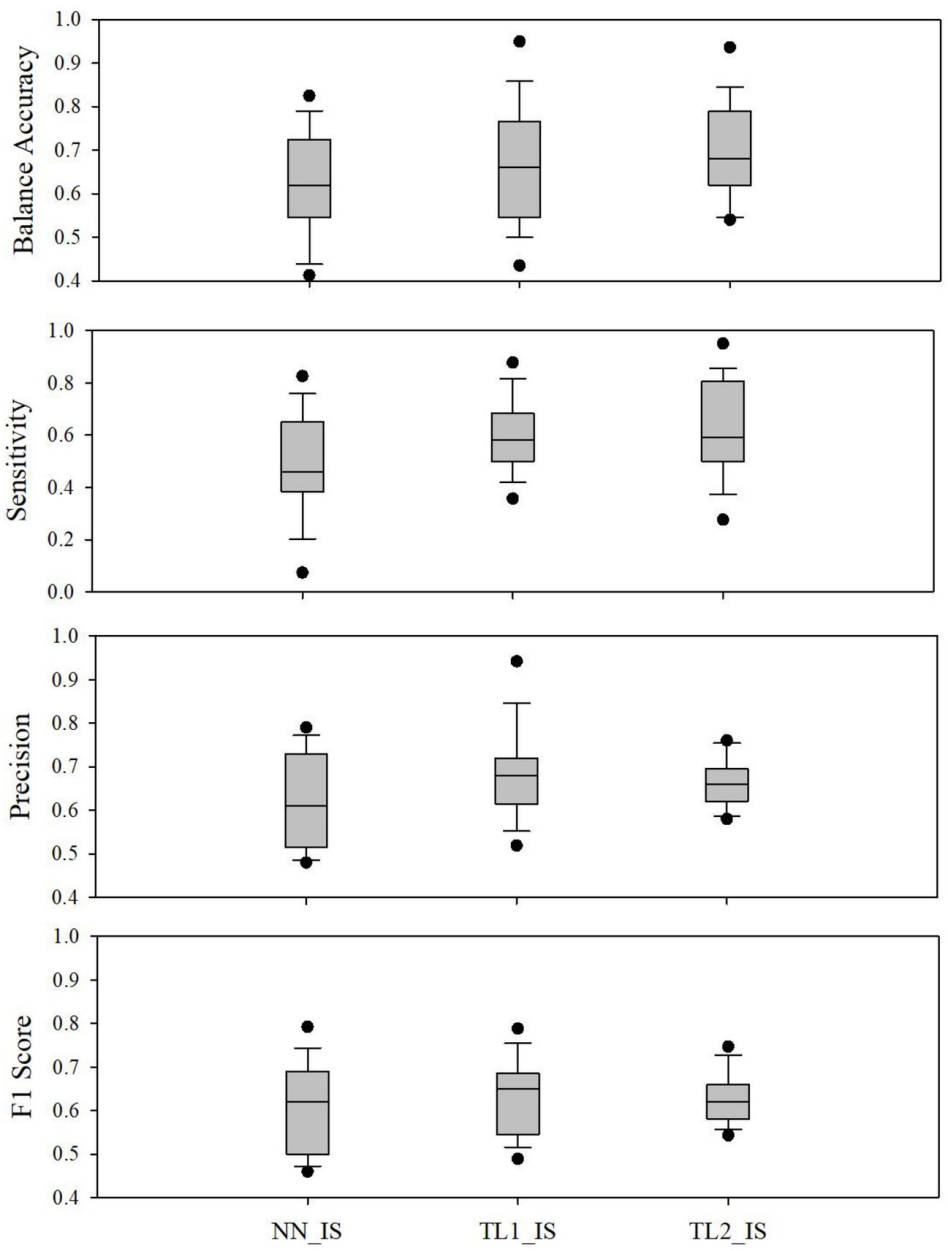
Comparison of performance results of a neural network model with imbalanced-sampling (NN-IS) and two transfer learning models with imbalanced-sampling (TL1-IS and TL2-IS).

Balanced accuracy is the average of a model’s success rate at classifying positive examples or negative examples as such. Sensitivity is the model’s success rate at classifying positive examples as positive. Technically, F_1_-score is the harmonic mean of sensitivity and another measure called “precision,” which measures the proportion of the times the model’s classification of a case as positive is actually correct. Therefore, F_1_-score is a complete measure of the model’s success at both correctly identifying high-risk cases and avoiding the incorrect classification of low-risk situations as high risk. We specifically described the models’ performance based on these three important measures; however, aggregate results are shown in Figs 4 and 5 for all measures.

### 3.2. Analysis of classification models

For each of the nine conventional machine learning techniques analyzed, we built two classifiers for each patient data—a model each with and without IS. The performance of these two approaches was estimated with data unseen to the models during training; the performances were compared to each other by comparing the average results on a five-fold cross-validation split. The aggregate results from these comparisons are presented in Fig 4.

Our selected final prediction techniques (TL1_IS or TL2-IS) achieved a balanced accuracy median (interquartile range) of 66(56~76) % for TL1_IS and 68(63~78) % for TL2_IS. Precision levels for TL1_IS and TL2_IS were 68(62~72) % and 66(62~69) % while sensitivity levels were 58(50~67) % and 59(51~80) % from 25 patients which were approximately 1.08 (accuracy, precision) to 1.28 (sensitivity) times increased in terms of performance outcomes, compared to NN_IS.

With regard to classification accuracy, our results established that with proper training and management of class imbalance in data, the median value of balanced accuracy for each machine learning model could reach approximately 60% or higher and K-NN showed median increase level after applying imbalance sampling (IS) method, compared to no IS, median (IQR) ratio (after/before) were highest [1.15 (1.08~1.32)] with KNN technique in the context of asthma exacerbation risk prediction (Fig 4; white boxes). For sensitivity, our results strongly demonstrated the importance of properly managing imbalanced data. The results showed that median sensitivities for models with the IS method increased by approximately 30% or higher for all models, compared to those values for models without IS technique (sensitivities of 10 to 40%. Similarly, we also found that the median values of Precision or F_1_-scores for each model were comparable or higher with the IS method (Fig 4).

For all of the performance measures, the aggregate performance statistics show generally increasing trends in the results of the conventional machine learning techniques. In particular, support vector machines, logistic regression and neural network performed well and the gains in weighted accuracy were 22.5%, 18.5%, and 8%, respectively. Their improvements in sensitivity were significant ranged from 145% to 467%.

### 3.3. Analysis of transfer learning

We evaluated the performance of two transfer learning models (TL1_IS and TL2_IS) and compared the results with those of a 1-layer neural network model with imbalanced sampling (NN_IS). Fig 5 shows the overall gains achieved in the transfer learning architectures over the neural network fully trained on only a target patient’s data.

For all of the performance measures, the aggregate performance statistics show generally increasing trends in the performance of NN_IS and the two TL models. TL1_IS showed 5.8% higher in weighted accuracy, 25.9% higher in sensitivity, and 11.4% in precision, compared to NN_IS. The performance improvements were more significant in TL2_IS for weighted accuracy and sensitivity. TL2_IS showed 8.5% higher in weighted accuracy, 28.3% higher in sensitivity, and 7.6% higher in precision, compared to NN_IS. It is worth mention that although these increases are relatively modest compared to what is achieved by transfer learning in other applications, such as image classification, they are achieved without the benefit of data augmentation commonly used for model improvement.

## 4. Discussion

The primary objective of this study was to improve the performance of machine learning techniques commonly applicable in public health or clinical researches to assess the impact of indoor air quality on PEFR values using clustered data of asthma patients. Among the models used, the highest performance, as evaluated by the median value of balanced accuracy, sensitivity, precision, and F1-score, was observed with the TL_IS models.

This study was conducted using daily data of indoor air quality and PEFR collected from adult asthma patients and their home environments. We observed that the room temperature was relatively stable at 20 to 22°C and the relative humidity ranged between 37–50%, which were relatively stable, unlike outdoor environment, thus our study results can be applicable to other indoor study conducted at asthmatic’s home.

Some studies have previously used machine learning techniques approached for the prediction of asthma exacerbation, including alarm systems, proposed by Lee et al. [29]; predictions of control deterioration, by Luo et al. [30]; feature extraction for risk detection, by Jalali et al. [31]; and machine learning frameworks for risk prediction, by Alkobaisi et al. [22]. Most of the previous studies still exhibit unsatisfactory accuracy for predicting the risk of asthma exacerbation in individual patients. A recent related work, proposed by Kim et al. [32], utilizes a deep learning model to predict asthma exacerbations among pediatric asthma patients with 70% precision for the prediction of the risk zone based on the EU asthma zoning scale [21]. Our work differentiates itself by utilizing an individualized-level risk zoning which is more accurate in comparison to public-level risk zoning as it is based on patients’ accumulated data. Moreover, our proposed transfer learning method has proven to further increase the prediction accuracy through two phases of the modeling the first is a population-level of data model followed by a second phase of model tuning to each individual.

To date, no study has systematically compared various models to assess their prediction performance. Our study provided results of comparative evaluation of performance improvement through an imbalanced sampling method for popular machine learning techniques in individualized asthma health risk prediction; our study was conducted using patients’ PEFR data, IAQ data of temperature, relative humidity, CO2 and PM2.5.

Deep learning has shown a great success in image classification but requires a large amount of data to train models for high quality of prediction. However, small sizes of individuals’ training data, make this a challenging task. Our proposed TL method shows promise in alleviating the challenges of small datasets. TL improves a model’s learning of a target patient’s data through the transfer of knowledge from a related task that has already been learned by source model using a larger group of patients’ datasets. It provides better results although our data size was just at the threshold of the level required to take full advantage of the power of the NN model architecture for asthma risk prediction. Furthermore, of note, the proposed TL models increased the weighted accuracy of the prediction techniques by approximately 5.5% to 11.8%, and the sensitivity by 14.4% to 26.9%, compared to neural network, which shows great promise in the prediction of individualized asthma exacerbation.

It is well established that personalized big data analysis and machine learning techniques can accurately predict asthma exacerbation or risk reduction, but the effort is still minimal. In this study, with the long-term PEFR data of 25 adult asthma patients matched with the real-time indoor air quality, including PM2.5 concentrations, we found that the predictive performance of each model can be different by model itself as well as duration of accumulation.

However, the following limitations of our study should be noted. The number of study participants (n = 25) is small and the number of samples was different for each patient. However, for each participant, the data were collected for a year, over 4 different seasons. This range ensures that the indoor air quality and their symptom distributions would not be systematically biased.

In the prediction of asthma exacerbation, we used two zones, a “red zone” nominally taken to be PEFR values in the lower 20% of the patient’s historical PEFR values and a “safe zone” which we nominally take to be PEFR values in the upper 80% of the PEFR distribution. The 80/20 cutoff here is somewhat arbitrary but following the EU Normal scale [21]. In practice, it would be important for doctors and patients together to analyze the patient’s PEFR distribution as it relates to his or her actual health condition and take care to modify the 80/20 cutoff as necessary. Additionally, the population data for source model in TL was approximately 3,800 (24 patients’ data), which is still small for deep learning classifiers. As the population size grows, TL may be the most promising models with regard for further improvements and implications in the future.

Our indoor air quality concentrations might not be representative of each sampling season or area as a result of spatial-temporal variations. Additional studies may be needed to understand the stability of our results in other databases collected from patients of other hospitals with a 1-year monitoring of indoor air quality.

Furthermore, in future studies, measurements of other risk factors of indoor air quality including concentration of biological allergic agent or molds, are expected to provide improved prediction performance. We used a real-time indoor air quality monitor, which should be checked for measurement error. It is well-known that the response of such real-time monitors with light scattering technique varies with aerosol size distribution, composition, and optical properties, and need proper calibration. We used our own calibration method as described previously [20]. In brief, before our field sampling in the homes of patients, we operated ESCORTAIRs and PAs simultaneously and checked measurement errors compared to federal equivalent methods.

In this study, the evaluation of the performances of the machine learning technology conducted with integrated data of daily indoor air quality collected through monitors from high QC/QA program as well as daily PEFR value. This makes this study unique compared to the previous studies, which were mostly conducted without such high-level QC/QA of indoor air quality monitors.

Transfer learning has been shown to significantly improve the accuracy of the target model for general image classification neural networks [28]. However, to date no such studies have been performed in the area of individualized asthma prediction with limited training data for each patient. To the best of our knowledge, this research is the first to propose an individual-based prediction method for asthma patients that is based on this deep learning technique of transfer learning and runs on indoor exposure data of asthma patients.

With continued innovation of machine learning technologies, its application to various database, especially clustered personal data, can flourish in the fields of clinical research and/or environmental health sciences. Transfer learning with imbalance sampling technique was best suitable for classification for adult asthmatics’ PEFR change according to the variation of indoor air quality with its high predictive accuracy among most available techniques. This study investigated only neural network-based transfer learning, and only one transfer learning strategy, freezing network layers and re-training the other layers for the target model. There are other strategies that could be tested for neural networks, including modifying the source model’s architecture before re-training, adjusting the level of fitting of the source model with regularization such as dropout or using the output of the middle layer of the source model to initialize a smaller target model such as support vector machine.
